# Growth patterns of lower limbs in children with a traumatic transtibial amputation: A retrospective observational cohort study of anthropometric data from Cambodia

**DOI:** 10.1097/PXR.0000000000000483

**Published:** 2025-10-14

**Authors:** Claudia Ghidini, Caitlin E. Edgar, Thearith Heang, Sisary Kheng, Anthony M. J. Bull

**Affiliations:** 1 The Centres for Paediatric Blast Injury Studies, Department of Bioengineering, Imperial College London, London, UK; 2 Exceed Worldwide and The Department of Prosthetics and Orthotics, Phnom Penh, Cambodia

**Keywords:** pediatric, transtibial, amputation, growth, growth plate, volume changes, children

## Abstract

**Background:**

The growth dynamics of the amputated limb are currently unknown for children, affecting prosthetic provision and child’s overall wellbeing.

**Objective:**

The objective of this study was to investigate the growth patterns of the residual limbs of children with a transtibial traumatic amputation.

**Study Design:**

A retrospective observational cohort study of anthropometric data from Cambodia was conducted using casefile of 19 children with a transtibial unilateral traumatic amputation (>100 visits).

**Methods:**

Anthropometrics of the lower limbs were correlated with age and limb length, and linear regression models were created to predict growth. Residual limb volume changes were estimated using a validated method.

**Results:**

The growth rate of the proximal epiphysis of the residual limb was found to be stunted and grew 33.3% slower than would have been expected for typically developing proximal epiphysis growth. This stunted growth can be accurately predicted using the known growth of the contralateral limb (adjusted R^2^ = 0.743, *p* < 0.0001, r = 0.864). Residual limb size (girth, bone condylar width) was correlated with age and limb length and can be predicted using linear regression models (girth: adjusted R^2^ = 0.665, *p* < 0.0001, r = 0.663 with age and r = −0.338 with limb length; tibial condyle width: adjusted R^2^ = 0.61, *p* < 0.0001, r = 0.784). Residual limb percentage volume changes between prosthetic replacements were above the 10% threshold used for prosthetic replacement in adult cohorts over 60% of the cases.

**Conclusions:**

This paper found that longitudinal residual limb growth is stunted because of growth plate injury and altered mechanics, paving the way for future research on novel rehabilitation interventions. The regression models developed can help prosthetists and prosthetic manufacturers design prostheses that accommodate growth. These data provide compelling evidence and hard data to prosthetists and manufacturers to better provide for children in this specific setting.

## Introduction

Lower limb loss in children poses unique and ongoing clinical challenges because of continuous physical growth, which complicates prosthetic management throughout childhood. Longitudinal (limb length) and circumferential (girth and volume) growth directly affect prosthetic fit,^1^ which in turn affects control, comfort, and functional mobility.^2^

Achieving and maintaining a well-fitting prosthesis is essential not only for effective control and comfort but also for residual limb and musculoskeletal health.^1,3^ In children, growth often leads to prosthetic tightness and discrepancies in length between the limb with prosthesis and intact limb.^1^ Specifically, residual limb growth can cause tightness of the socket, which is the interface between the residual limb and the rest of the prosthesis, resulting in residual limb conditions^1^ and potentially leading to prosthetic disuse.^4^ Progressive relative shortening between the residual limb and the intact limb, because of the loss of the distal growth plate in trans-osseous amputation, exacerbates issues such as prosthetic length discrepancy (where the prosthesis becomes shorter than the intact limb).^1^ In addition, it results in a shorter lever arm of the residual limb at skeletal maturity, which is critical for prosthetic control and may impair the user’s ability to effectively use a prosthesis.^5-8^

In adults, poor socket fit and prosthetic length discrepancy have been associated with secondary impairments resulting from compensatory movements (e.g., relying on the intact limb more during the stance phase of gait). These compensatory movements are adopted to reduce pain^2^ and maintain function, but they increase the incidence of other conditions such as osteoarthritis of the intact joints, low back pain, scoliosis, or osteoporosis/osteopenia of the residual limb bones.^2,9,10^ In children, these issues are likely to be further exacerbated by their continuous physical growth. Unlike adults, children undergo rapid longitudinal and circumferential growth, which quickly renders a once-well-fitted prosthesis inadequate.^11^ Current prostheses for children have limited adjustability, partially because of the lack of information on residual limb growth, and cannot account for these significant physical changes.^11-13^ Therefore, to avoid issues related to poor fit and maintain function, these changes must be closely monitored throughout childhood.^3^ This increases the frequency of clinical visits and prosthetic replacements, placing a considerable burden on children and healthcare providers.^1,3,6,13^ However, frequent replacements are not always feasible, especially in low-resource settings. Prolonged use of a poorly fitting prosthesis in a growing child may have significant long-term consequences on the immature musculoskeletal system, which responds more readily and dramatically to altered loads (such as seen in cerebral palsy).^14^ This represents a critical global challenge, particularly for children with limited or inconsistent access to prosthetic care.

Despite this, there is currently no research on the longitudinal growth of the residual limb, how it compares to the intact side, and the maximum prosthetic length discrepancy experienced by this cohort. Although longitudinal residual limb growth may be expected to follow a predictable pattern such as a proportional reduction based on the known contribution of the missing distal growth plate^3,7,15^ (43% for distal tibial growth plate^16^), this would fail to account for inhibitory factors such as initial injury, altered blood supply and mechanical environment that might result in stunted growth.^5,17^ Furthermore, although the maximum acceptable threshold of prosthetic length discrepancy has been defined as 5 mm for adults with a transtibial amputation,^9^ no such threshold exists for the pediatric population—despite the likelihood that children may experience far greater discrepancies over time.

In addition, there is no research that has investigated residual limb volume changes in children with lower limb loss.^18^ In adults, it has been found that marginal volume changes, such as 5%–10%, cause discomfort, and may necessitate socket replacement to alleviate pain.^19,20^ Similarly, data are unavailable for the anthropometric parameters key to prosthetic limb suspension and socket manufacturing in children with a transtibial amputation, such as girth of the residual limb and tibial and femoral condylar width.

The aim of this study is to investigate the circumferential and longitudinal growth patterns of the residual limbs of children with a transtibial traumatic unilateral amputation, and to relate this to the growth of intact limbs. A linear growth assumption was made as growth charts for typically developing children show a quasi-linear relationship until 14–15 years of age,^21^ and growth of the tibia is fairly uniform throughout childhood.^22^

The following hypotheses are tested, that the

1. growth of the remaining proximal growth plate in the residual limb is reduced compared to a typically developing plate resulting in reduced longitudinal growth;
2. girths of the residual limb are correlated with chronological age and limb length and thus can be predicted using multilinear regression model;
3. femoral and tibial condylar widths of the residual limb are correlated with chronological age and can be predicted using a linear regression model;
4. residual limb percentage volume changes between prosthetic replacements in this cohort are significant and frequently higher than the 10% threshold used for prosthetic replacement in adults; and
5. prosthetic length discrepancy in the children exceeds the 5 mm threshold used in adults.

## Methods

This study was approved by the National Ethics Committee for Health Research of Cambodia (NECHR_24) and the institutional ethics committee in the United Kingdom (ICREC_6609720). The research study was performed in accordance with the Declaration of Helsinki. Individual participant consent and legal guardian consent were waived because the study used anonymized clinical data. As this is a retrospective analysis of an existing dataset, no patient was involved in the study design.

Nineteen casefiles of children who received a unilateral, traumatic transtibial amputation were manually hand scanned across 3 prosthetic centers of the Department of Prosthetics and Orthotics in Cambodia. Information contained in the casefiles was reported in a spreadsheet to allow detailed analysis. Cases of congenital deficiencies were not included in this study as they are often extremely complex, and limb and bone morphology significantly deviate from typical.^23^ Snakebite cases were not included as growth may be stunted because of venom, meriting a separate analysis.^24^ Only unilateral cases were included to enable comparison to the contralateral, intact limb.

Demographics, including sex, age at amputation, and age at first clinical access, were analyzed using descriptive statistics.

Seventeen out of 19 casefiles included multiple visits (from 2007 to 2023) where the anthropometrics of the lower limbs were collected (Figure 1). Anthropometric measurements are collected by prosthetists using a tape measure and an anthropometer whenever children require a new prosthesis. Measurements collected for the residual limbs are used to make the prosthetic socket and include girths at different levels of the residual limb starting from the patellar tendon (PT) as well as skeletal measurements of femoral and tibial medio-lateral (M-L) widths (Figure 1(a)). Measurements collected for the intact limb include length of the shank from tibial plateau to base of heel, as well as the biggest and smallest girths of the shank; these measures are used by the prosthetists to make cosmetic covers (Figure 1(b)).

**Figure 1.**
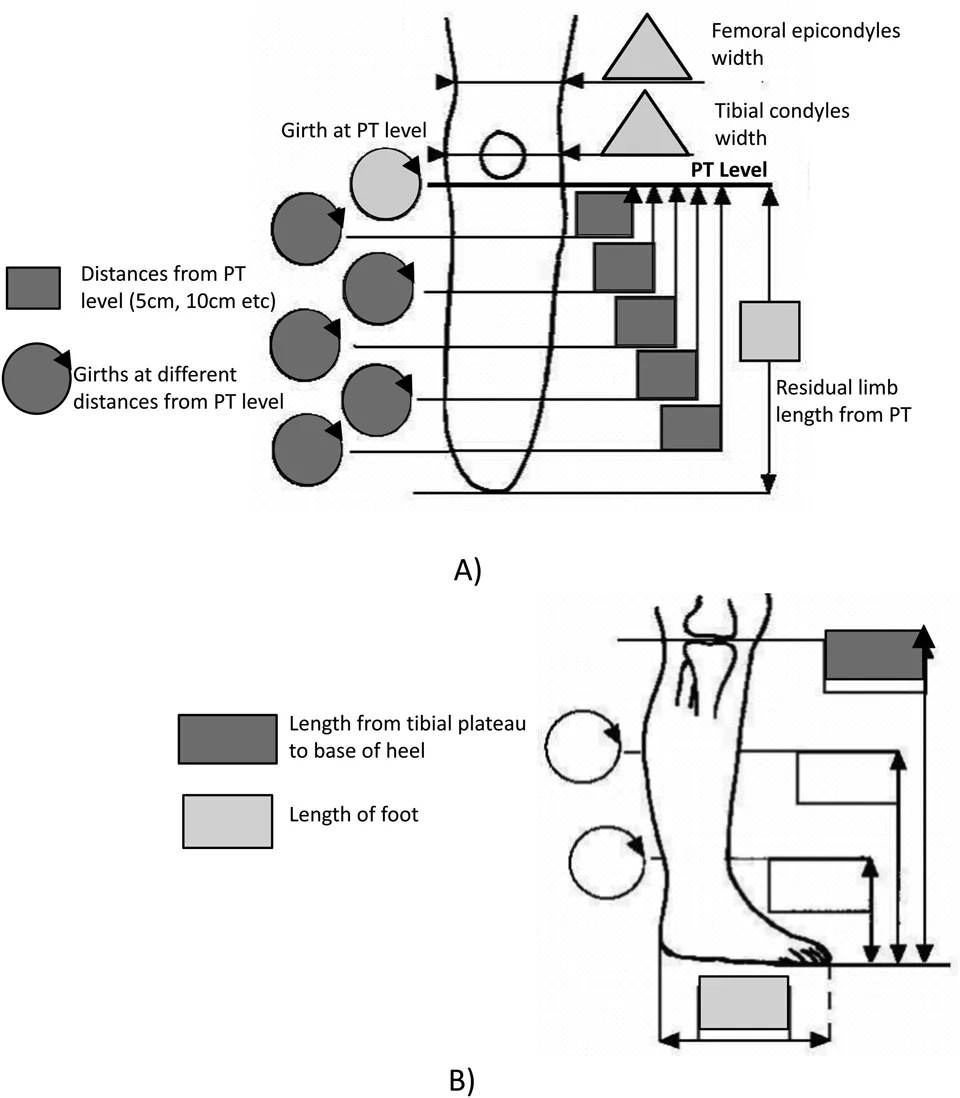
Anthropometric measurements collected for (a) residual transtibial limb; (b) intact limb.

In this study, the terms “visit” only refers to a clinical appointment where a new prosthesis is fabricated, as this is when anthropometric measurements are collected. Children also attend the prosthetic center for reasons other than growth such as prosthetic repairs (e.g., loose screws) or pain (e.g., socket’s adjustments to relieve pressure).

### Assumption testing

To test the assumption of tibial growth linearity, a Pearson correlation analysis was conducted between length of the intact shank (from tibial plateau to heel) and with chronological age, with significance set at *p*-value of 0.05. Linear regression was then conducted.

### Hypothesis 1: reduced longitudinal growth of the residual limb

The intact tibial length was modeled as the sum of the initial length plus the increases because of the proximal and distal growth plates separately (Equation 1). Literature values regarding the contribution of the proximal and distal growth plates are known and based on cadaveric estimation from typically developed children^16^ and are reported in Figure 2.^25^ These values represent the proportional contributions of the growth plates and can be used with the assumption of tibial growth linearity to produce Equation 2.^7,22^ The increase in intact shank length between visits is calculated using Equation 3.

(1) $$L_{i_{t}}\left(t\right)=L_{i_{0}}+C_{P}\Delta_{i_{t}}+C_{D}\Delta_{i_{t}}$$

(2) $$L_{i_{t}}\left(t\right)=L_{i_{0}}+0.57\Delta_{i_{t}}+0.43\Delta_{i_{t}}$$

(3) $$\Delta_{i_{t}}=L_{i_{t}}\left(t\right)-L_{i_{0}}$$

$L_{i_{t}}$ → length of the intact shank at given time *t*

$L_{i_{0}}$ → first measurement of intact shank length

$\Delta_{i_{t}}$ → increase in length of intact shank

$C_{D}$ → Contribution of distal growth plate

$C_{P}$ → contribution of proximal growth plate

**Figure 2.**
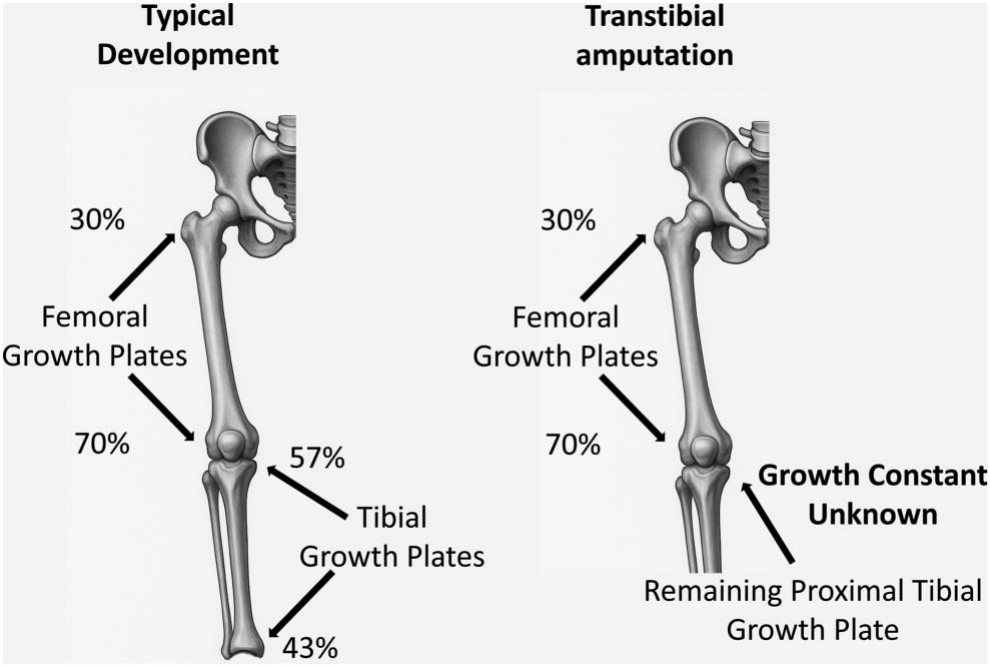
Comparison of the growth potential of a typically developing lower limb and a limb affected by transtibial amputation, values from Cunningham et al.^16^ Image created with ChatGPT.^25^

The residual limb growth model was derived as shown in Equations 4–7 using the increase in length of the intact limb ($\Delta_{i_{t}}$). First, the loss of the distal growth plate is accounted for by removing its contribution (removal of 0.43 $\Delta_{i_{t}}$) (Equation 4). Second, the altered mechanical and physiological environment postamputation is considered through an unknown constant *C*_*A*_ (Equation 5).

Equation 7 is found by combining Equations 5 and 6. A linear regression model was fitted to the data $\Delta_{r,t}$ (difference in residual limb length, Equation 6) using the predicting variable $\Delta_{i,t}$ (difference in intact limb length).

(4) $$L_{r_{t}}\left(t\right)=L_{r_{0}}+0.57\Delta_{i_{t}}$$

(5) $$L_{r_{t}}\left(t\right)=L_{r_{0}}+0.57{C_{A}\Delta }_{i_{t}}$$

(6) $$\Delta_{r_{t}}=L_{r_{t}}\left(t\right)-L_{r_{0}}$$

(7) $$\Delta_{r_{t}}={0.57C}_{A}\Delta_{i_{t}}$$

$L_{r_{t}}$ → length of the residual limb at given time t

$L_{r_{0}}$ → first measurement of residual limb length

$\Delta_{i_{t}}$ → increase in length of intact shank

$C_{A}$ → constant to account for altered mechanical and physiological environment

$\Delta_{r_{t}}$→ increase in length of residual limb

The percentage change in rate of growth between typically developing and residual limb proximal plate was estimated using Equation 8.

(8) $$\%\;\text{change}\;\text{in}\;\text{proximal}\;\text{growth}\;\text{rate}=\frac{{0.57C}_{A}-C_{P}}{C_{P}}*100$$

### Hypotheses 2 and 3: girth and width of residual limbs

To test hypothesis 2, Pearson correlation between girths of residual limb and age and girth and distance from PT was calculated using the 432 residual limb girth measurements collected in the casefiles. A multilinear regression model was created.

To test hypothesis 3, Pearson correlation was calculated for the M-L widths between femoral epicondyles and tibial condyles against chronological age and linear regression models were created.

### Hypothesis 4: residual limb volume changes over time

The volume of the residual limb was estimated using a cylindrical method of sections, validated for adults with a transtibial amputation.^26,27^ The limb was divided into sections, and the volume of each section estimated (Figure 3, Equations 9 and 10) and summed (Equation 11).^26^ As the distal circumference of the residual limb is usually not documented, the volume for the final section of the residual limb was estimated as a constant diameter cylinder using the value of the most distal girth collected as C in Equation 10. *V*_tot_ was calculated for each prosthetic provision instance across all participants.

(9) $$C=\frac{C_{i}+c_{i}}{2}$$

(10) $$V_{i}=\pi h_{i}{\left(\frac{C}{2\pi }\right)}^{2}$$

(11) $$V_{\text{tot}}=\sum V_{i}$$

**Figure 3.**
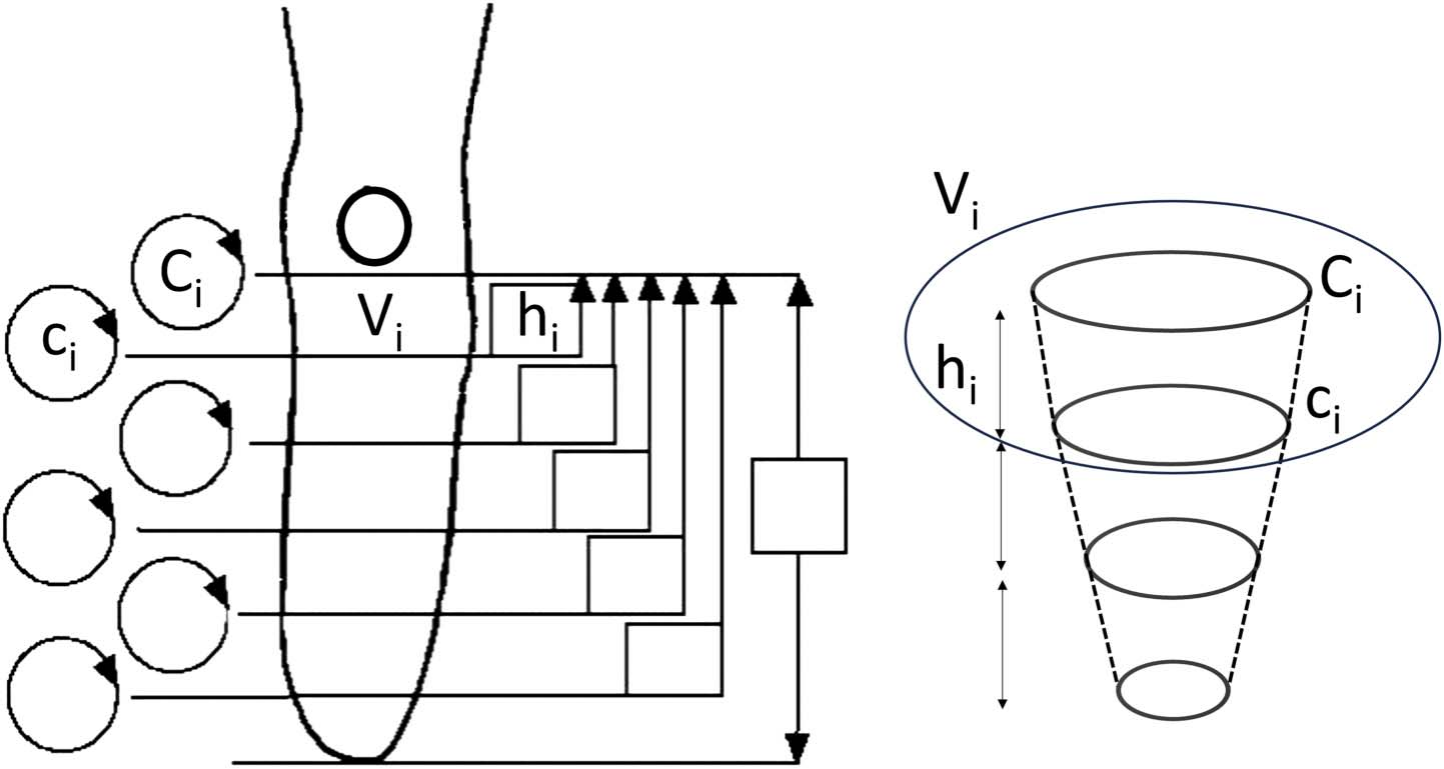
Anthropometrics used to estimate volume of sections of the limb are adjacent circumferences and section height. The individual volumes are then summed to obtain an estimate of the residual limb volume.

$C_{i}$ → proximal circumference of each section

$c_{i}$ → distal circumference of each section

C → mean of adjacent circumferences

$h_{i}$ → length of each section

$V_{i}$ → volume of each section

$V_{\text{tot}}$ → volume of the limb

The percentage volume changes were calculated and normalized to account for different limb sizes and age groups and allow comparison. The normalization was to the number of years passed between 2 successive prosthetic replacements ($t_{i+1}-t_{i}$) (Equation 12).

(12) $$\text{Yearly}\;\%\;\text{volume}\;\text{changes}=\frac{V_{\text{tot}}\left(t_{i+1}\right)-V_{\text{tot}}\left(t_{i}\right)}{V_{\text{tot}}\left(t_{i}\right)}*\frac{100}{t_{i+1}-t_{i}}$$

Only casefiles with at least 2 visits could be included in this analysis, resulting in the analysis of percentage volume changes for 17 out of 19 casefiles and 110 visits. All data points were analyzed to identify potential outliers (method described below), and each set of data was analyzed in detail. Reasons for exclusion were

1. significant weight loss,
2. revision surgery (residual limb shortening), and
3. significant volume shrinkage after amputation (first 3 months after surgery).

In total, 9 visits were excluded from the residual limb volume analysis.

The threshold of 10% for adult prosthetic replacement is used to test hypothesis 4.

### Hypothesis 5: prosthetic length discrepancy

The prosthetic length discrepancy for the cohort was estimated averaging the prosthetic length discrepancy for the visits where it was collected (7/32). Standard deviation was also estimated.

The threshold of 5 mm for adult prosthetic replacement is used to test hypothesis 5.

### Statistical analysis

All the regression models were created in MATLAB (R2022a, MathWorks), and their predictive power was evaluated following the same methods.

First, the normal probability plot of the regression standardized residuals was analyzed to check that the residuals are normally distributed, to ensure that the assumptions for use of a linear regression model are met.^28^ Then, the scatter plot of the standardized residuals of the model fit was analyzed to determine if there were any outliers with a large influence,^29^ defined as standardized residual greater than ±3.3 and with a Cook distance >1.^28,29^

Pearson correlation coefficient (r) and *p*-value of correlation with significance set at 0.05 was calculated for each comparison. Thresholds for strong (r ≥ 0.50), medium (0.3 ≤ r < 0.5), and small (0.1 ≤ r < 0.3) correlation were defined according to the literature.^28^

The linear regression models were evaluated with the adjusted R^2^ value, the root mean square error (RMSE), and the *p*-value with significance set at 0.05.^30^ An adjusted R^2^ value above 0.7 shows a good fit of the model to the data.^28,30^ A RMSE <2.50 cm was defined as acceptable based on practical limitations of hand measurement using a tape measure.

Depending on normality, a Wilcoxon Signed Rank test or a paired-samples *t* test was used to identify statistically significant differences in the rate of growth of the proximal plates in different conditions (typically developing growth plate vs. residual limb growth plate).

## Results

Approximately 68.4% (13) of the 19 children were male. The mean age at amputation was 8.6 years (±4.6), and the mean age at first clinical access was 10.3 years (±4.4).

A total of 112 visits where a new prosthesis was made (initial fitting or replacement) and anthropometric data collected was analyzed. Approximately 8.9% of the visits related to the first prosthetic provision, 34.8% of the prosthetic replacement visits were solely because of growth, and 17.0% because of growth and other issues such as breakage of components. The reported growth problems for each visit are detailed in Table 1. The remaining visits (40.2%) were because of components breakage, revision surgery, and others. The mean time between successive prosthesis replacement was 11.0 ± 8.5 months, and the number of visits per child ranged from 1 to 16 (5.9 ± 4.5 visits per child).

**Table 1.**
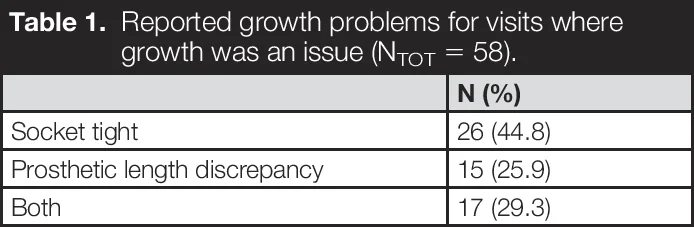
Reported growth problems for visits where growth was an issue (N_TOT_ = 58).

	N (%)
Socket tight	26 (44.8)
Prosthetic length discrepancy	15 (25.9)
Both	17 (29.3)

Two data points were missing for tibial condylar width, whereas 1 data point was missing for both intact shank length and femoral condylar width.

### Assumption testing

The intact shank length (Y) is linearly correlated with age (X) (*p* < 0.0001; Figure 4) with a good fit (adjusted R^2^ = 0.823) and correlation coefficient of 0.908. The RMSE of 2.72 cm is above the threshold set.

**Figure 4.**
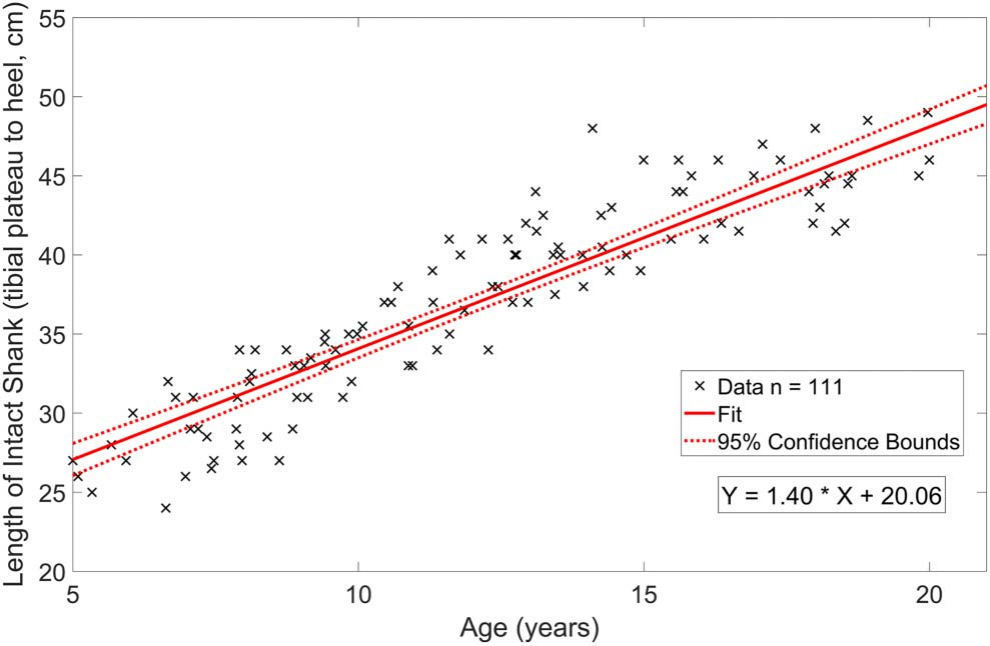
Scatterplot of regression of intact shank length (from tibial plateau to heel, Y) and age of participants (X), (black). Linear fit model for the dataset (red) with 95% confidence bounds (red dotted).

### Hypothesis 1: reduced longitudinal growth of the residual limb

Longitudinal growth of the residual limb (Y) is linearly correlated with longitudinal growth of the intact shank (X) (*p* < 0.0001; Figure 5) with a good fit (adjusted R^2^ = 0.743) and correlation coefficient of 0.864. The RMSE of 1.18 cm is below the threshold set.

**Figure 5.**
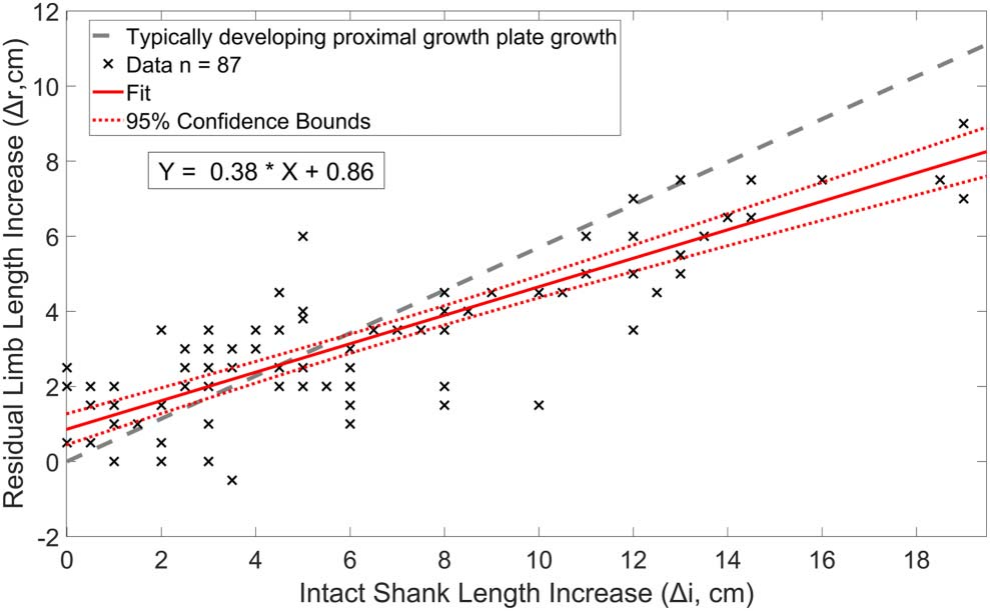
Residual limb length increases at a lower rate than predicted based on typically developing plate growth. Longitudinal growth of the residual limb (Y) is linearly correlated with longitudinal growth of the intact shank (X). Linear fit model and 95% confidence bounds are plotted in red (red solid and red dotted line). Each cross is one casefile observation (black, n = 87). Gray dashed line represents the contribution to growth of a typically developing proximal growth plate to enable visual comparison of the different rates.

The percentage decrease in growth rate between residual limb proximal growth plate and typically developing growth plate is 33.3%, and Wilcoxon Signed Rank test found a statistically significant reduction in growth rate for the remaining proximal growth plate of residual limb compared to typically developing one (*p* = 0.003), with a large effect size (r = 0.46).

### Hypotheses 2 and 3: girth and width of residual limbs

Girth of the residual limb (Y) can be correlated with age (X_1_) as well as distance from the PT (X_2_) using multifactorial regression (*p* < 0.0001; Figure 6) with a good fit (adjusted R^2^ = 0.665) and correlation coefficient of 0.663 for age and girth and −0.338 for girth and distance from PT. The RMSE of 2.78 cm is above the threshold set.

**Figure 6.**
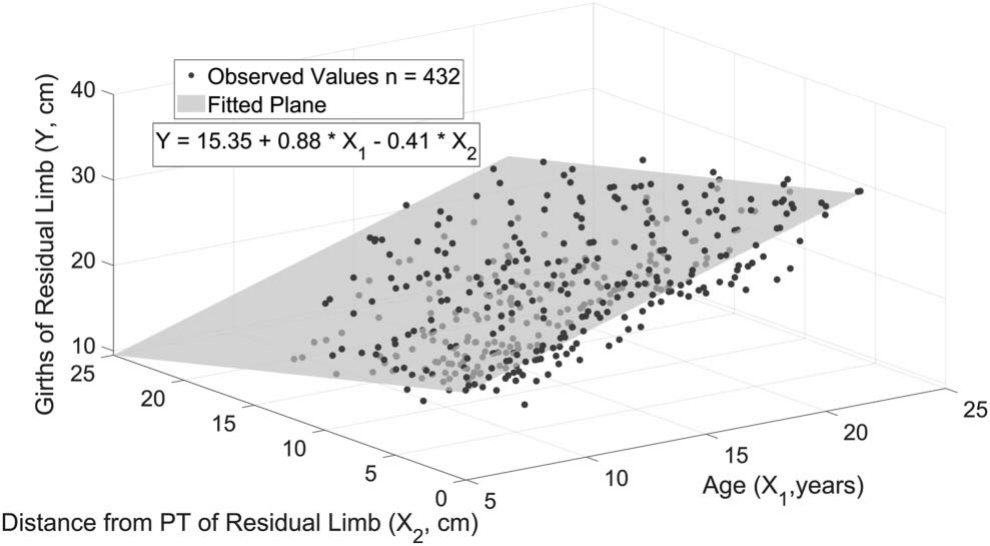
Multifactorial regression to predict girth of the residual limb (Y) based on age (X_1_) and distance from PT level (X_2_).

The tibial condyles M-L width (Y) is linearly correlated with age (X) (*p* < 0.0001) with a moderately good fit (adjusted R^2^ = 0.61) and correlation coefficient of 0.784. The RMSE of 0.78 cm is below the threshold set. The model formula is Y = 0.23X + 5.20.

The femoral condyles M-L width (Y) is linearly correlated with age (X) (*p* < 0.0001) with a moderately good fit (adjusted R^2^ = 0.472) and correlation coefficient of 0.691. The RMSE of 0.91 cm is below the threshold set. The model formula is Y = 0.21X + 3.67.

### Hypothesis 4: residual limb volume changes over time

Yearly % volume changes are reported in order of magnitude in Figure 7, overlayed with the threshold recommended for prosthetic replacement in the adult cohort (±10%^19^). In 68.3% of the visits after initial prosthetic provision and included in the volume analysis (58 out of 85 visits), growth was the cause of the prosthesis replacement.

**Figure 7.**
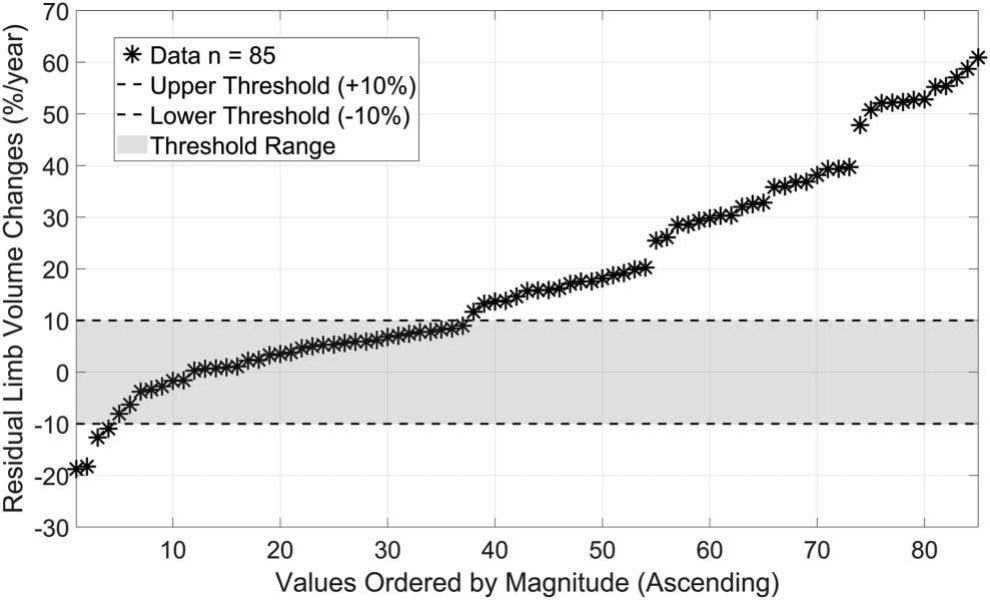
Percentage residual limb volume changes normalized by the time between visits for children with a transtibial amputation. Data are presented in order of magnitude (x-axis—values ordered by magnitude).

In only 38.8% of the cases were the volume increases within the acceptable clinical threshold for a prosthetic replacement.

### Hypothesis 5: prosthetic length discrepancy

The prosthetic length discrepancy recorded when replacing the prosthesis ranged from 10.0 to 20.0 mm, with average 14.3 ± 4.5 mm. In all cases where this measurement was available, the discrepancy exceeded the 5.0 mm threshold commonly applied in adult populations.^9^

## Discussion

All 5 hypotheses in this study were proven. Intact limb longitudinal growth was confirmed to be consistent with typical developmental trajectories.^22^ However, for the first time, it was shown that the growth rate of the proximal epiphysis of the residual limb is stunted and can now be accurately predicted using the increase in length of the contralateral limb. This stunted growth may be due mechanobiological effects such as because of initial trauma and subsequent surgery^17^ or the altered mechanical loading.^31^ If the former, investigations into differing growth patterns by cause of injury and subsequent surgical practice merit attention. In addition, further investigations to consider interventions harnessing biological pathways and mechanical loading to maximize residual limb length at skeletal maturity to support better prosthetic control are warranted.^5,6,8^

The girths of the residual limb are correlated with chronological age and limb length and thus can be predicted using multilinear regression model; femoral and tibial condylar distances of the residual limb are correlated with chronological age and can be predicted using a linear regression model. These findings provide valuable quantitative inputs that can inform socket and prosthetic fabrication and design. Specifically, the models allow clinicians and prosthetists to anticipate future changes in limb dimensions and incorporate this foresight into their decision making. For example, sockets can be fabricated using flexible thermoplastic materials, modular liners, or expandable panels that accommodate increases in limb girth without necessitating immediate replacement.^11,15^ Prosthetic systems can also integrate adjustable pylons or telescopic components to allow for straightforward lengthening as the child grows, thereby prolonging functional use and reducing the need for frequent replacements.^32^ Beyond immediate clinical applications, these models also assist the development of novel prosthetic designs and technologies tailored for growing children. Engineers can leverage these data to create next-generation pediatric prostheses with built-in adaptability—such as smart materials that respond to growth stimuli, 3D-printed components that can be easily reconfigured, or modular designs that can be expanded or reshaped over time.

It was also found that in most cases, residual limb percentage volume changes between prosthetic replacements in this cohort are above the 10% threshold used for prosthetic replacement in the adult cohort.^19,20^ This is the first time such data are reported and highlights the prevalent challenge children face where they often must rely on poorly fitting sockets for extended periods before seeking replacements, potentially compromising their comfort and increasing the likelihood of residual limb conditions or other secondary impairments.^1,2^ Similarly, prosthetic length discrepancies at the time of replacement were consistently above the adult threshold of 5 mm. Despite the limited number of data points for this subanalysis, these findings highlight the need for further studies on pediatric-specific clinical criteria for prosthetic adjustment and replacement. Importantly, these findings also reinforce the need to prioritize the design and implementation of prosthetic components with built-in adjustability—such as expandable sockets or telescopic pylons^11,15,32^—that can accommodate predictable growth and reduce the frequency of full prosthesis replacements.

There are several limitations of this research study. First, the study is retrospective, and there were some missing data points, which may have affected the accuracy of the predictions. In addition, the use of age as a sole predictor for growth, rather than more comprehensive measures such as height and weight, introduces potential limitations in the predictive models employed. Moreover, the utilization of chronological age rather than skeletal age may further limit the accuracy of growth predictions, as previous research suggests that skeletal age may be a more reliable indicator of growth trajectories.^33^ The inclusion of data from both male and female subjects, analyzed collectively because of insufficient sample sizes for each sex, may have obscured potential sex-specific trends in growth dynamics, which merits further investigation given their known variations.^22^

Second, the measurement methods were limited. For example, the method employed to calculate residual limb volume, although validated, was not a full surface scan (such as could be achieved with 3D optical scanners or medical imaging). Also, the girths were not collected in the same way for every visit as there were some visits for which different numbers of circumferences were collected along the residual limb. The lengths collected during the clinical appointment refer to the full limb length (full residual limb length from PT to distal end, length of intact shank from tibial plateau to the base of the heel) and not the bone itself, which would have been a more appropriate skeletal measure. There were multiple clinicians taking the measurement, leading to potential inaccuracies.^34^ Repeated measures from the same participants were not accounted for, which could have increased biases and goodness of fit. A key limitation of this analysis is the reliance on adult-derived thresholds for volume change and prosthetic length discrepancy, which may not accurately capture the impact of poorly fitting prostheses on the child’s developing musculoskeletal system; establishing appropriate, age-specific thresholds represents an important direction for future research.

Finally, the study data are from Cambodia only, which may constrain the generalizability of findings to broader populations of children with transtibial amputation. Further studies incorporating larger, more diverse datasets and greater longitudinal follow-up is warranted to corroborate these findings. Generalizing to other levels of amputation is not possible without access to datasets of children with transfemoral and through-knee amputations.

In conclusion, this paper is the first to identify the stunted longitudinal growth of the residual limb, caused by trauma and altered mechanical environment, which paves the way for future research into strategies to mitigate this (e.g., rehabilitation interventions or surgical approaches). The novel models developed, predictive of residual limb size, can be used by prosthetists and prosthetic manufacturers to design prostheses that can accommodate for growth and reduce the necessity for clinical access, which is especially relevant for children living in humanitarian settings where access to care is often limited.

## Acknowledgments

The authors wish to thank the incredible clinical and administrative teams at all three prosthetic centres in Cambodia for maintaining such detailed clinical casefiles.

## Funding

The author(s) disclosed receipt of the following financial support for the research, authorship, and/or publication of this article: support from Save the Children UK and the UK Engineering and Physical Sciences Research Council grant EP/S02249X/1 for the Centre for Doctoral Training in Prosthetics and Orthotics. This fundings supported the study design, data collection, analysis, and interpretation of data, as well as writing of the manuscript.

## Declaration of conflicting interest

The author(s) disclosed no potential conflicts of interest with respect to the research, authorship, and/or publication of this article.

## Data availability statement

Data are available on 10.5281/zenodo.14187212.
